# Supplementary material for: Unsupervised inter-domain transformation for virtually stained high-resolution mid-infrared photoacoustic microscopy using explainable deep learning
Source: Nat Commun. 2024 Dec 30;15:10892. doi: 10.1038/s41467-024-55262-2 (PMC11685655; doi:10.1038/s41467-024-55262-2)
Supplement: Supplementary file 1 — Supplementary Information [file 41467_2024_55262_MOESM1_ESM.pdf]

Supplementary information for:

Unsupervised inter-domain transformation for virtually stained high-resolution mid-infrared photoacoustic microscopy using explainable deep learning

Eunwoo Park[1,2,§], Sampa Misra[1,2,§], Dong Gyu Hwang[2,3,§], Chiho Yoon[2,4], Joongho Ahn[2,4], Donggyu Kim[1,2], Jinah Jang[1,2,3,6,7,8,*], and Chulhong Kim[1,2,4,5,6,7,9,*]

[1]*Department of Convergence IT Engineering, Pohang University of Science and Technology (POSTECH), Pohang, Republic of Korea*

[2]*Medical Device Innovation Center, Pohang University of Science and Technology (POSTECH), Pohang, Republic of Korea*

[3]*Center for 3D Organ Printing and Stem Cells, Pohang University of Science and Technology (POSTECH), Pohang, Republic of Korea*

[4]*Department of Electrical Engineering, Pohang University of Science and Technology (POSTECH), Pohang, Republic of Korea*

[5]*Opticho Inc., Pohang, Republic of Korea*

[6]*Department of Mechanical Engineering, Pohang University of Science and Technology (POSTECH), Pohang, Republic of Korea*

[7]*Department of Medical Science and Engineering, Pohang University of Science and Technology (POSTECH), Pohang, Republic of Korea*

[8]*Institute for Convergence Research and Education in Advanced Technology, Yonsei University, Seoul, Republic of Korea*

[9]*Graduate School of Artificial Intelligence, Pohang University of Science and Technology (POSTECH), Pohang, Republic of Korea*

[§]These authors contributed equally to this work

[*]Corresponding authors: Chulhong Kim (chulhong@postech.edu) and Jinah Jang (jinahjang@postech.ac.kr)

Table of contents

Supplementary Notes

Supplementary Note 1. Specifications of the MIR-PAM system 3
Supplementary Note 2. XDL-MIR-PAM of living cells 11

Supplementary Figures

Supplementary Fig. 1. XDL-UIDT network architecture 2
Supplementary Fig. 2. Imaging performance of the MIR-PAM system 3
Supplementary Fig. 3. XDL-IREN-generated images and salience masks according to the training epochs 4
Supplementary Fig. 4. GradCAM heatmap in each transformer layer of the XDL-based generator 5
Supplementary Fig. 5. Verification for the HR-MIR-PAM 6
Supplementary Fig. 6. Visual comparison of frameworks by network combination 7
Supplementary Fig. 7. Biological feature extraction from XDL-MIR-PAM duplexed images 8
Supplementary Fig. 8. Magnified images of XDL-UDIT 9
Supplementary Fig. 9. XDL-MIR-PAM of fibrotic HCFs 10
Supplementary Fig. 10. XDL-MIR-PAM of living HCFs 12
Supplementary Fig. 11. XDL-UIDT performance according to the noise variances 13

Supplementary Tables

Supplementary Table 1. Four-fold cross-validation result 14

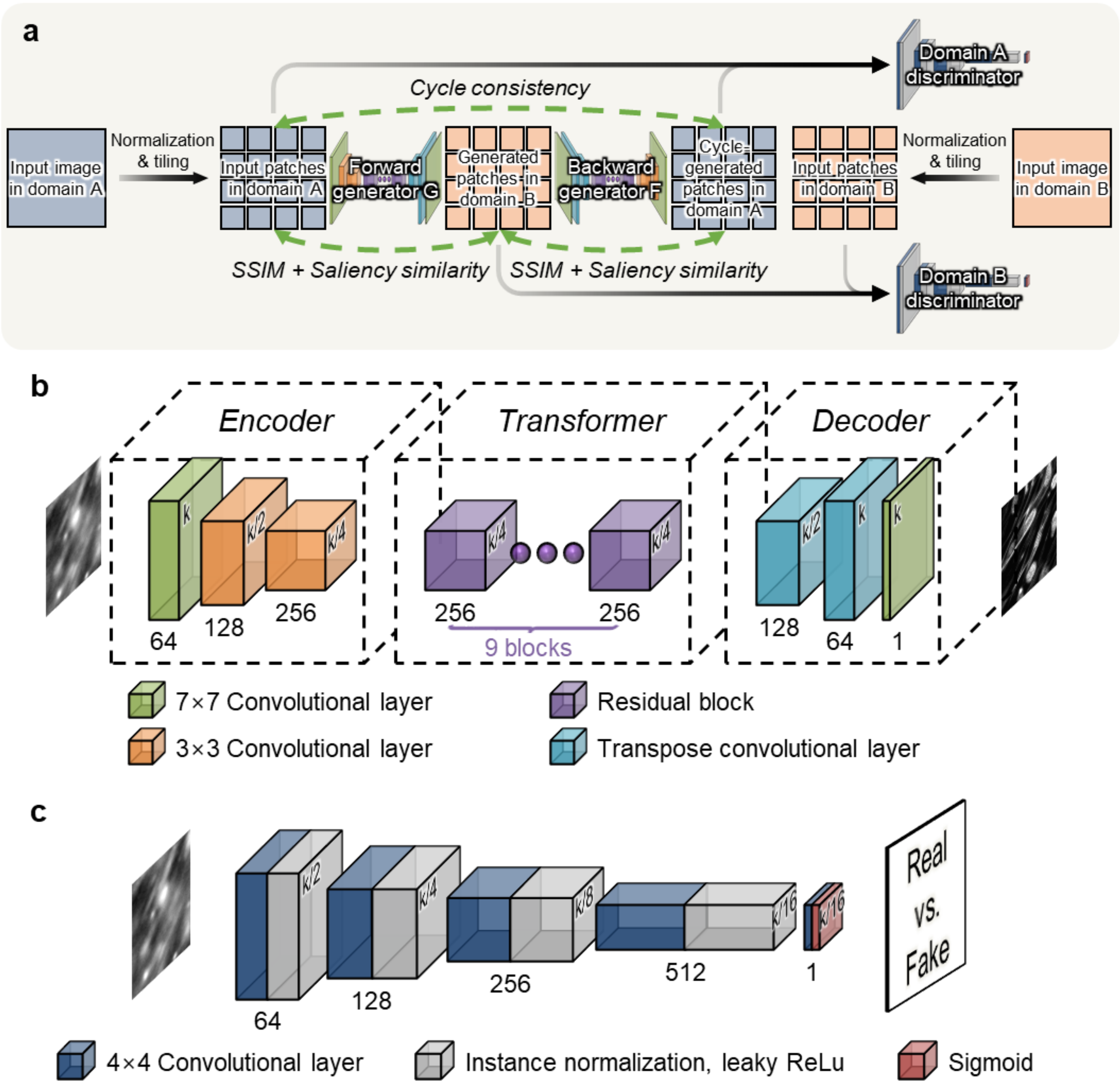

Supplementary Fig. 1 | XDL-UIDT network architecture. a, Schematic diagram of XDL-based CycleGAN. **b,** Generator. **c,** Discriminator.

Supplementary Note 1 | Specifications of the MIR-PAM system

To evaluate the imaging performance of the MIR-PAM system, a 5 µm wide gold pattern printed film was photoacoustically imaged in heavy water at a wavelength of 6.00 µm to determine the spatial resolutions. The lateral resolution was measured to be 6.6 µm, close to the theoretical value of 6.12 µm. The axial resolution was measured to be 57.5 µm, where the theoretical value is 55.2 µm when the speed of sound in heavy water is assumed to be 1380 m/s [1]. Both spatial resolution values were calculated by the full width at half maximum (FWHM) of each profile envelope. In addition, we found an imaging depth of 60.7 µm at which the PA signal intensity diminished by -6 dB using a sloped surgical suture. The laser power irradiated to the sample was 0.2 mW.

For HCF imaging (on day 7), the signal-to-noise ratio (SNR) and contrast-to-noise ratio (CNR) were 26.48 and 23.02, respectively. In detail, the peak-to-peak voltage levels of the signal, background, and noise were 27.8 mV, 3.63 mV, and 1.05 mV, respectively (with 200 A-line averages).

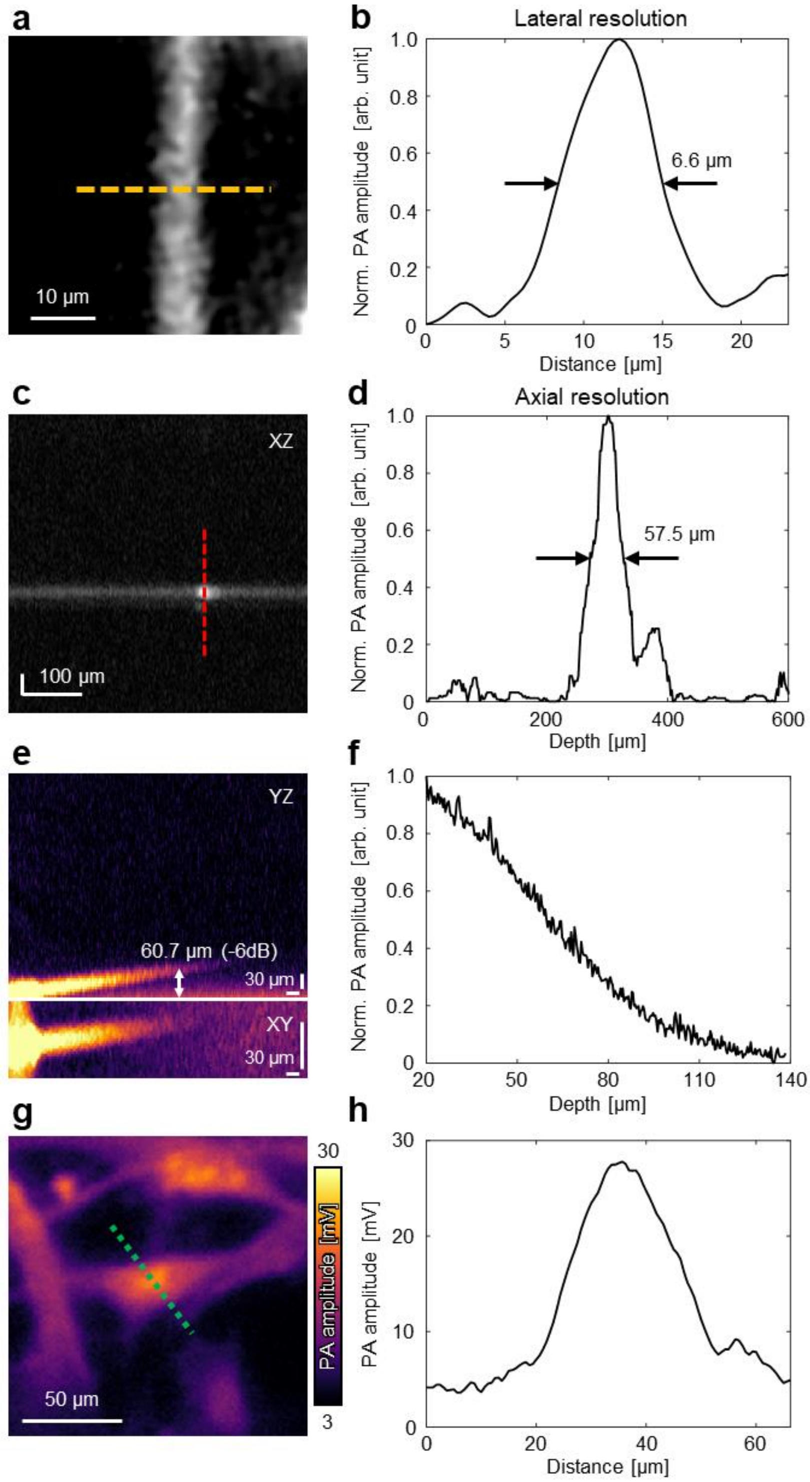

Supplementary Fig. 2 | Imaging performance of MIR-PAM system. a, PA maximum amplitude projection (MAP) image of a gold pattern printed film. **b,** Line profile along the orange line in **a** for the lateral resolution. **c,** B-scan PA image of a surgical suture. **d,** Line profile along a red line in **c** for the axial resolution. **e,** PA MAP images of a sloped surgical suture. **f,** Normalized PA signal amplitude along the central depth of the suture. **g,** PA MAP image of cultured HCF. **h,** PA line profile at the green dotted line in **g**. Source data are provided as a Source Data file.

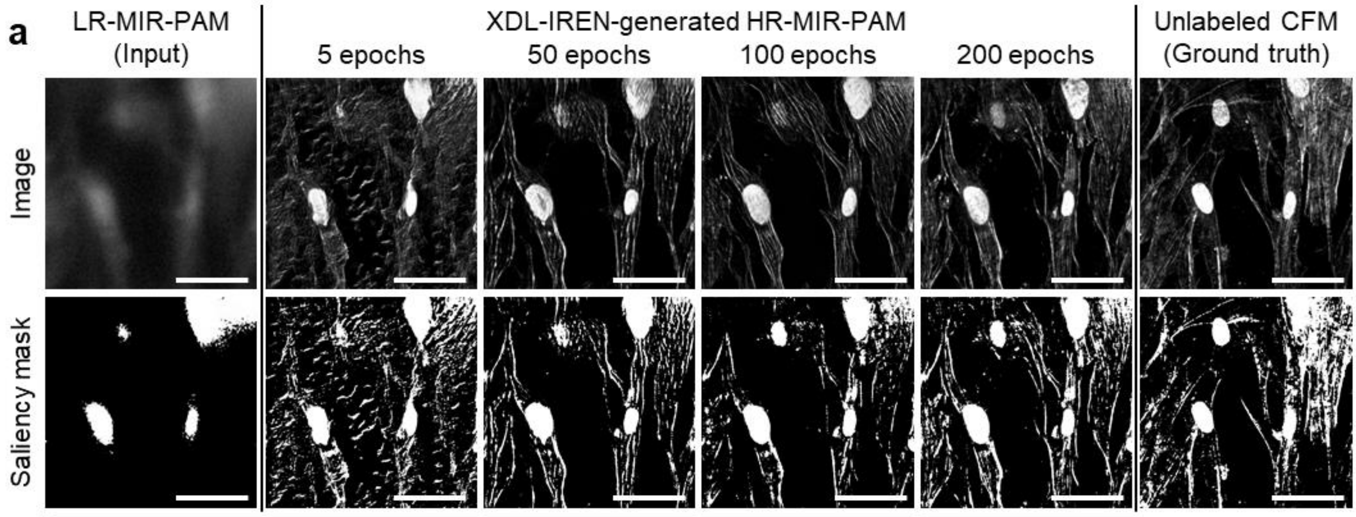

	5 epochs	50 epochs	100 epochs	200 epochs
FID (↓)	331.21	262.10	146.05	125.87
KID (↓)	29.60	19.43	8.81	4.78

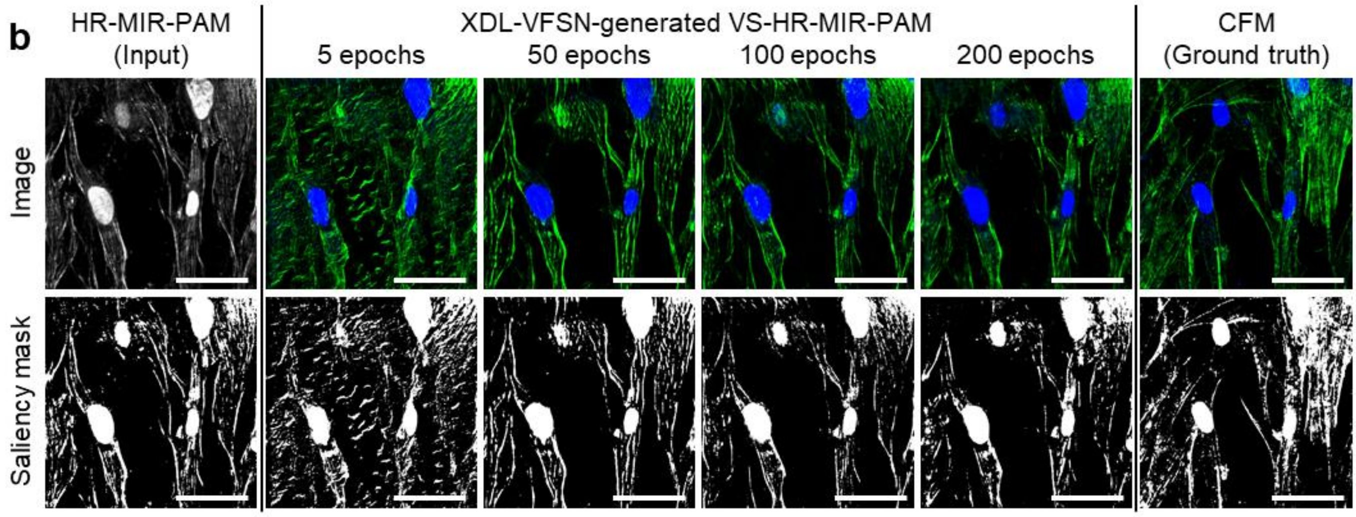

	5 epochs	50 epochs	100 epochs	200 epochs
FID (↓)	182.85	104.32	101.17	88.01
KID (↓)	15.90	5.04	4.73	3.06

Supplementary Fig. 3 | XDL-generated images and saliency masks in a. IREN and b. VFSN according to the epochs. Scale bars, 50 μm.

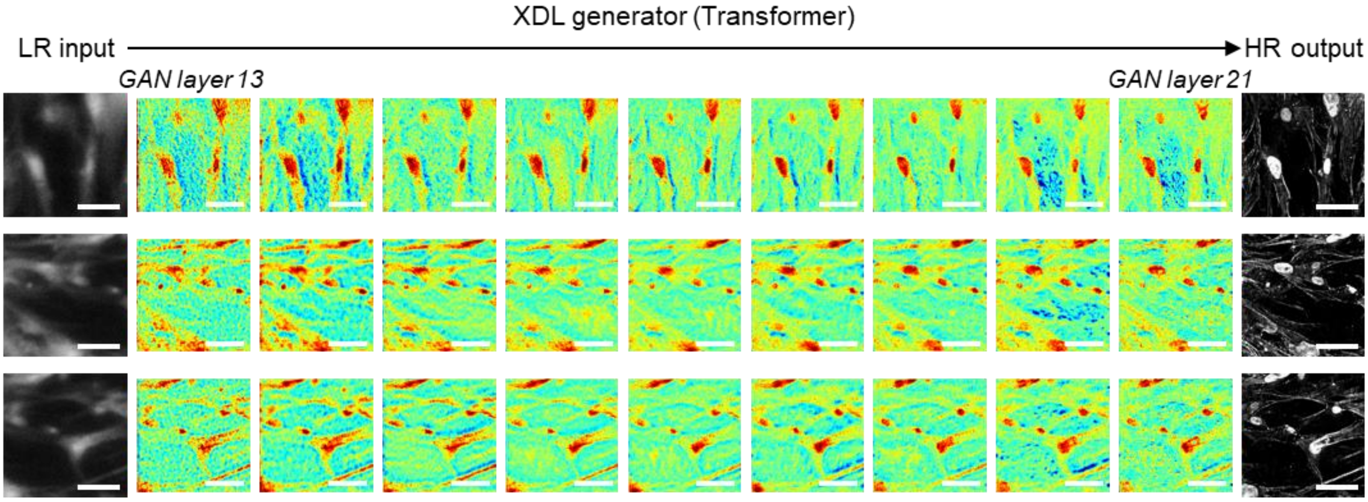

Supplementary Fig. 4 | GradCAM heatmap in each transformer layer of the XDL-based generator. Scale bars, 50 μm.

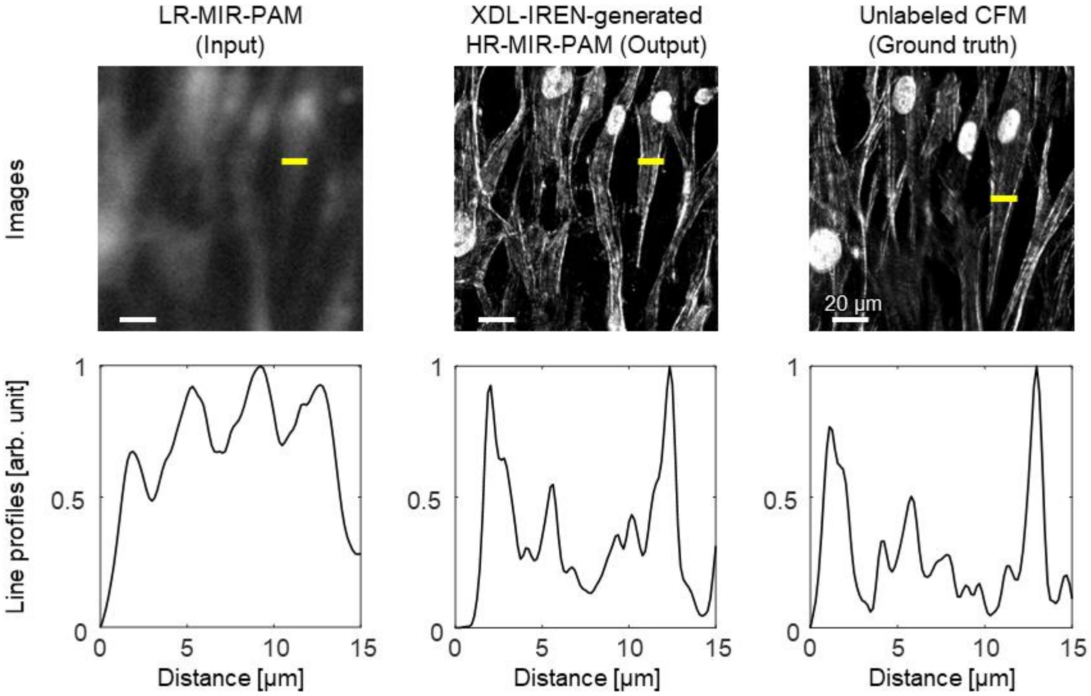

Supplementary Fig. 5 | Verification for the HR-MIR-PAM. For quantitative evaluation of the XDL-IREN, line profiles along the yellow lines of each corresponding image are compared. Scale bars, 20 μm. The XDL-IREN-generated HR-MIR-PAM images capture detailed structures of HCF (1–2 μm) beyond the resolution of LR-MIR-PAM (6–7 μm). Source data are provided as a Source Data file.

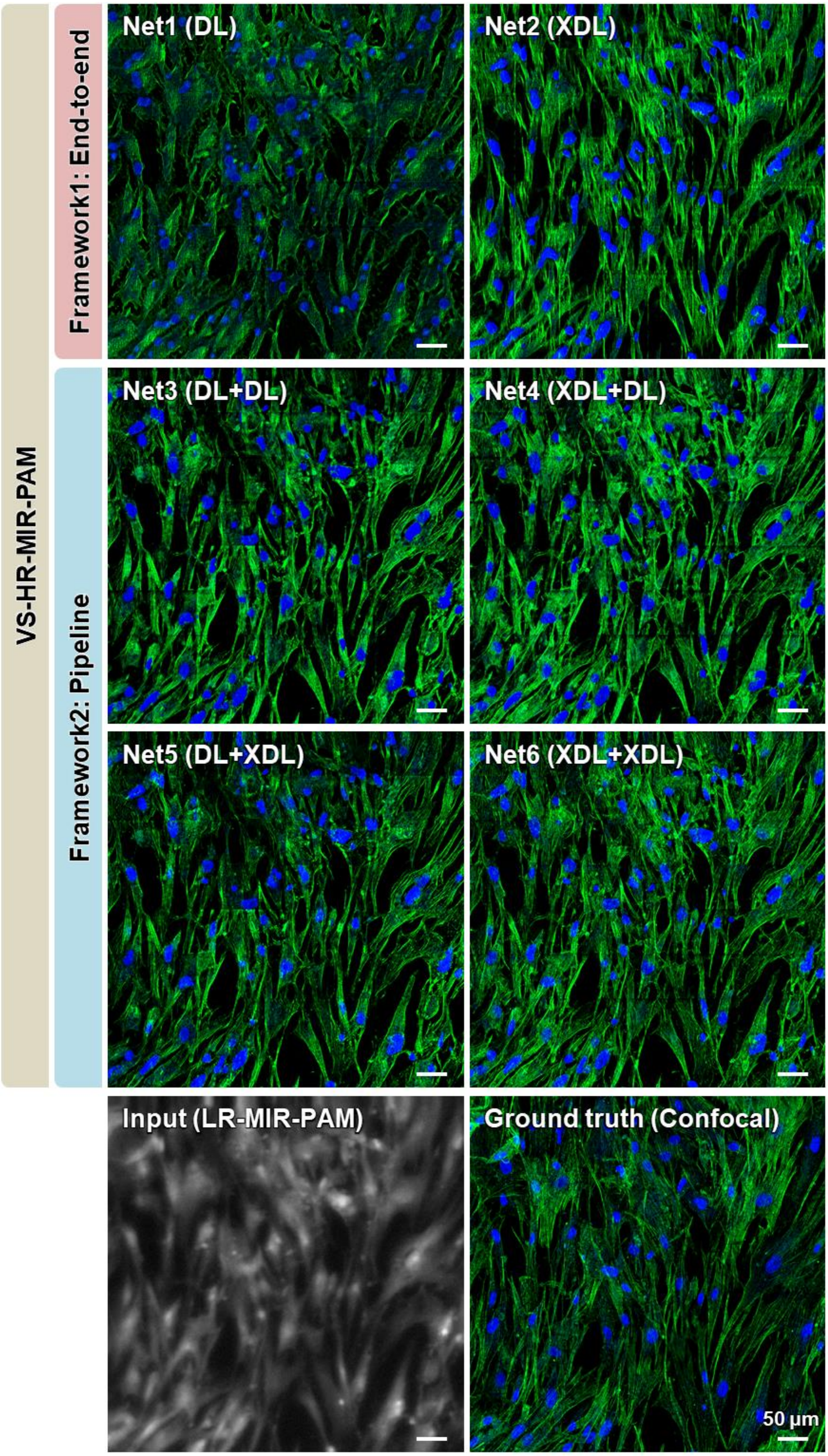

Supplementary Fig. 6 | Visual comparison of frameworks by network combination.

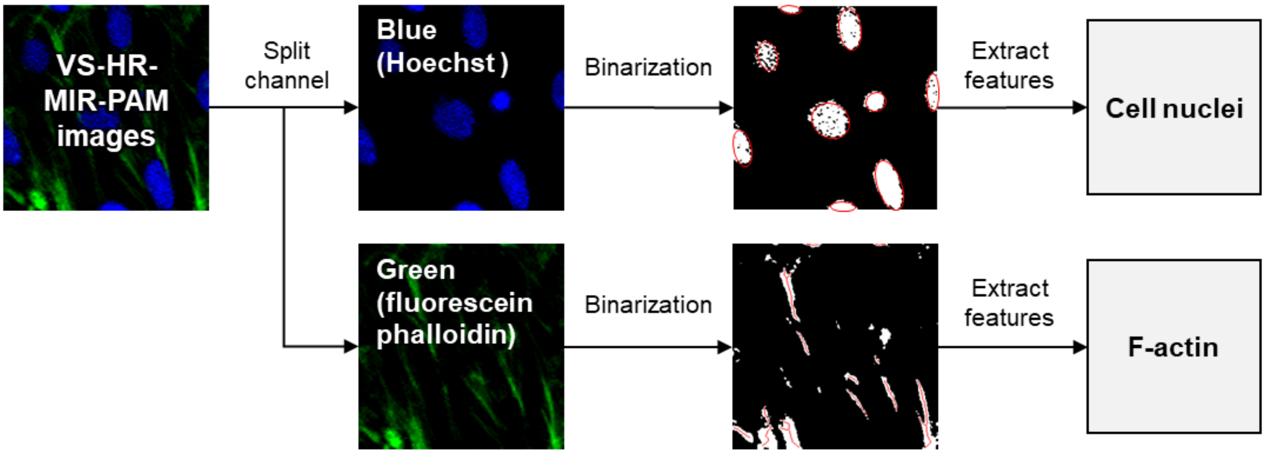

Supplementary Fig. 7 | Biological feature extraction from XDL-MIR-PAM duplexed images.

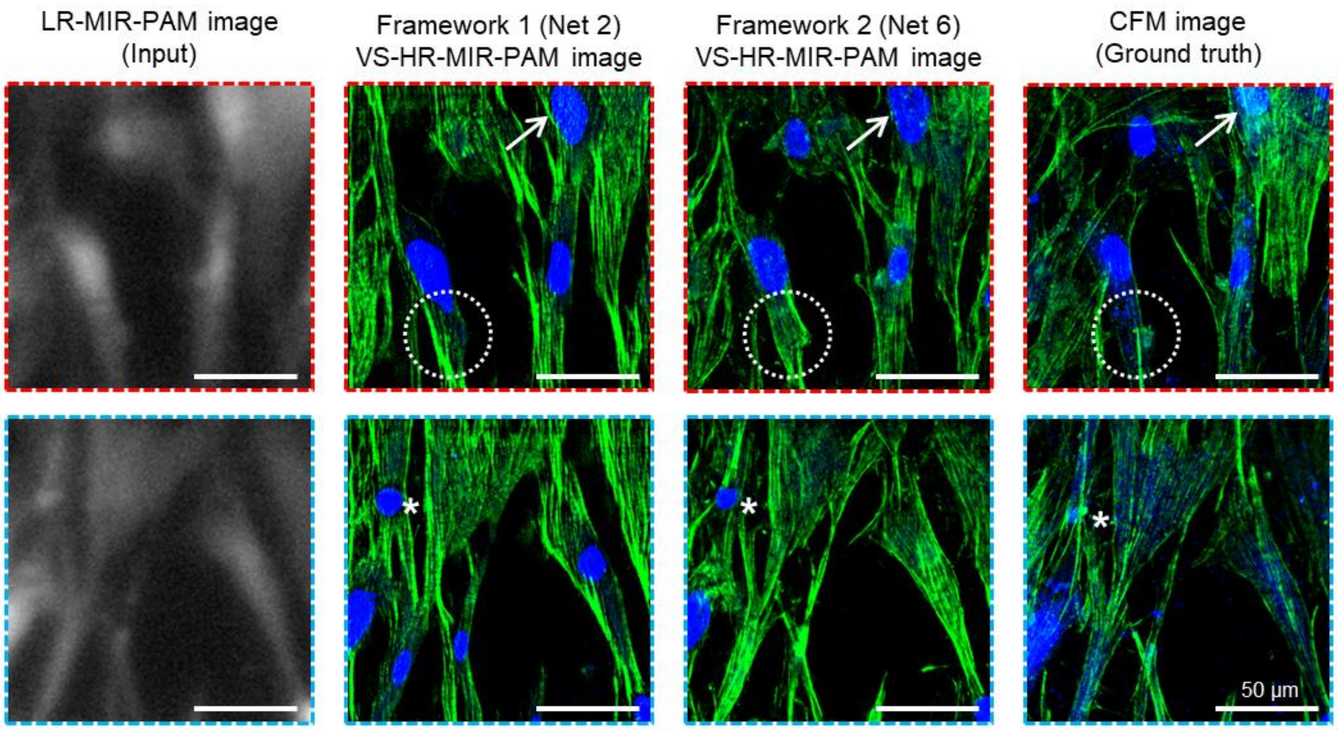

Supplementary Fig. 8 | Magnified images of XDL-UDIT. Scale bars, 50 μm.

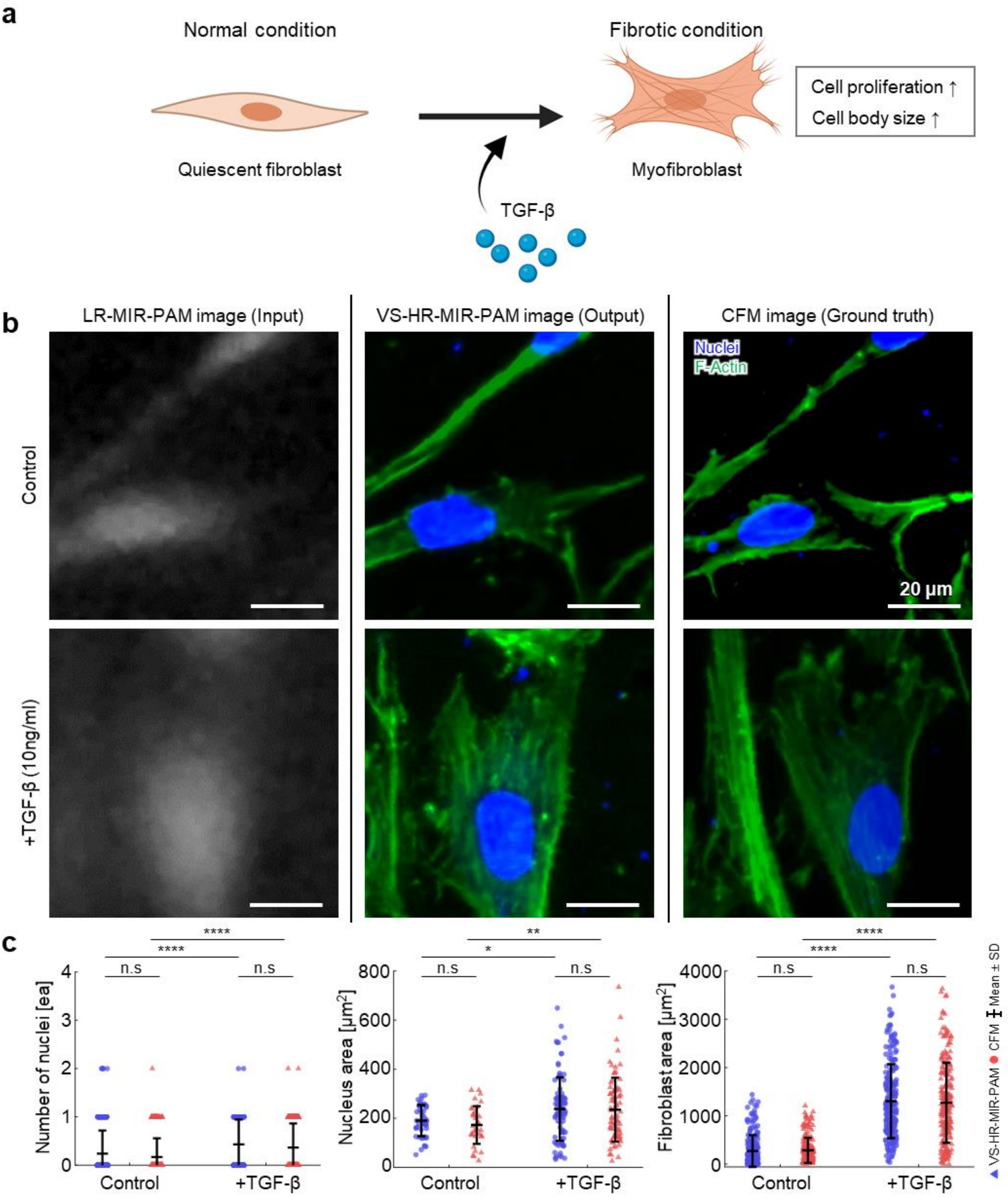

Supplementary Fig. 9 | XDL-MIR-PAM imaging of fibrotic HCFs. a, Conceptual schematic of activation. (Created in BioRender. Kim, M. (2024) https://BioRender.com/j39a961). **b,** Visual comparison of XDL-MIR-PAM images between the domains. Scale bars, 20 μm. **c,** Comparisons of biological features according to cell growth: Number of nuclei, nucleus area, and fibroblast area (n = 225). Significance by two-way ANOVA with uncorrected Fisher's LSD test: n.s, not significant ($p > 0.05$), *, $p < 0.005$, and ****, $p < 0.0001$. Source data and p-values are provided as a Source Data file.

Supplementary Note 2 | XDL-MIR-PAM of living cells

While traditional CFMs have struggled with photobleaching and phototoxicity, XDL-MIR-PAM can overcome these problems by implementing resolution enhancement and virtual staining in label-free living cells. The feasibility of MIR live-cell imaging is confirmed by the cell viability under MIR laser irradiation [2]. In the same way as fixed cells, LR-MIR-PAM images and CFM images of HCF were used as the input and ground truth, respectively. Supplementary Fig. 8a depicts a strategy for the live-cell XDL-MIR-PAM. To generalize XDL-UIDT, both fixed and living cell image sets are included in the training dataset. In particular, only images of days 1 and 7 were trained and targeted to generate XDL-MIR-PAM images of living HCF on days 1, 4, and 7. We adopted the pipelined framework (Net 6) and tested it to generate the VS-HR-MIR-PAM images. Supplementary Fig. 8b shows the results of XDL-MIR-PAM of living cells according to HCF growth. On days 1, 4, and 7, LR-MIR-PAM images were obtained and VS-HR-MIR-PAM images were generated and compared with corresponding CFM images. The overall HCF confluency increases over the days. The F-actin structures that were indistinct and difficult to identify in the LR-MIR-PAM input images are predicted in detail and appear as fibers in the VS-HR-MIR-PAM images on all days. Supplementary Fig. 8c quantifies the change in the biological features. In the VS-HR-MIR-PAM images, the number and area of the cell nuclei do not vary significantly over the days. In contrast, the fibroblast area increases by 67.6% overall. It increased sharply to 42.2% in the early phase (day 1–4), and slowly to 17.8% in the late phase (day 4–7). These metrics show strong correlations ($R^2 > 0.95$) with values in CFM. The XDL-MIR-PAM accurately predicts every stages of living cell growth.

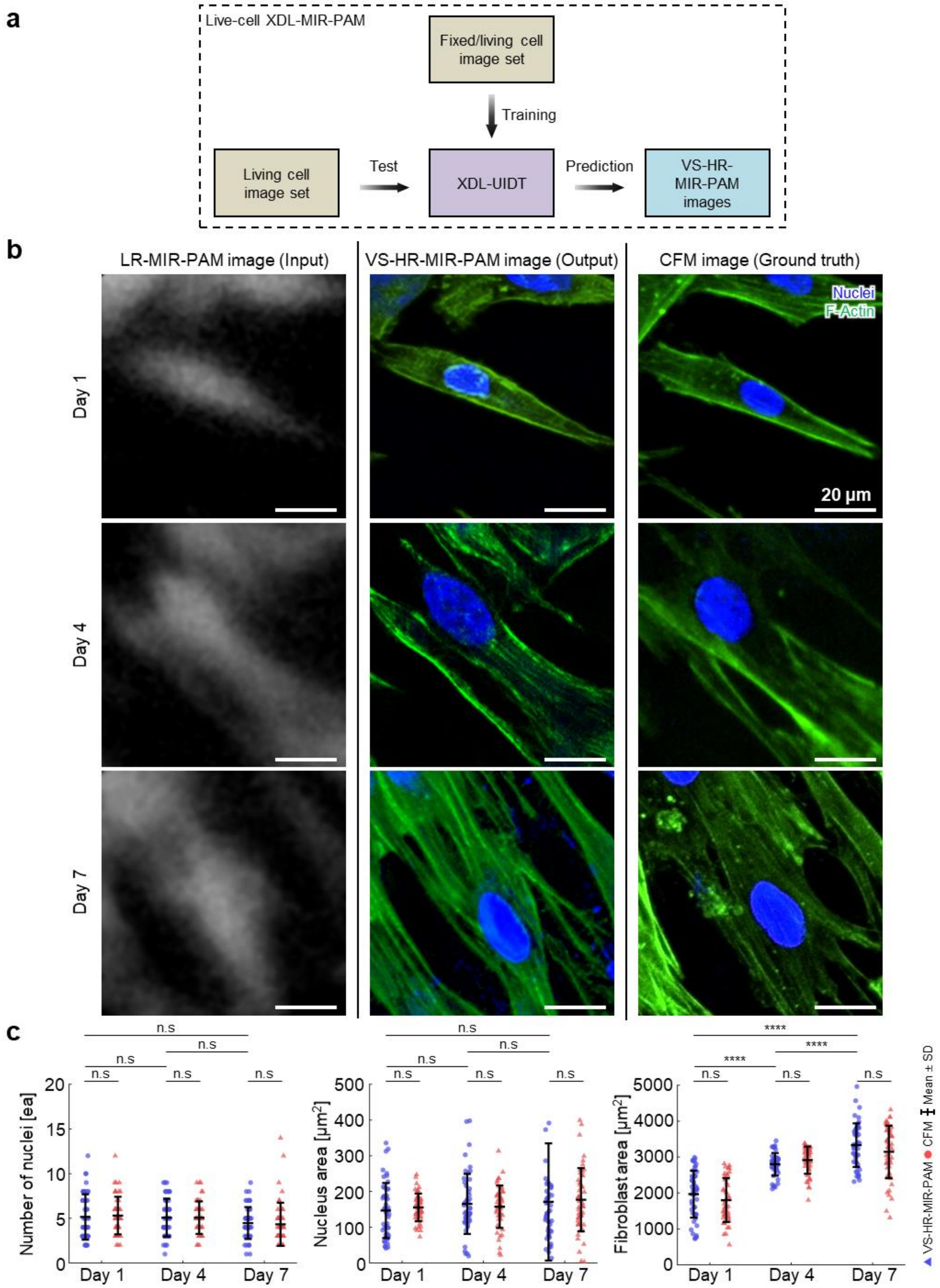

Supplementary Fig. 10 | XDL-MIR-PAM imaging of living HCFs. a, Conceptual workflow of live-cell XDL-MIR-PAM. **b,** Visual comparison of XDL-MIR-PAM images between the domains. Scale bars, 20 μm. **c,** Comparisons of biological features according to cell growth: Number of nuclei, nucleus area, and fibroblast area (n = 49). Significance by two-way ANOVA with Šídák's multiple comparisons test: n.s, not significant ($p > 0.05$) and ****, $p < 0.0001$. Source data and p-values are provided as a Source Data file.

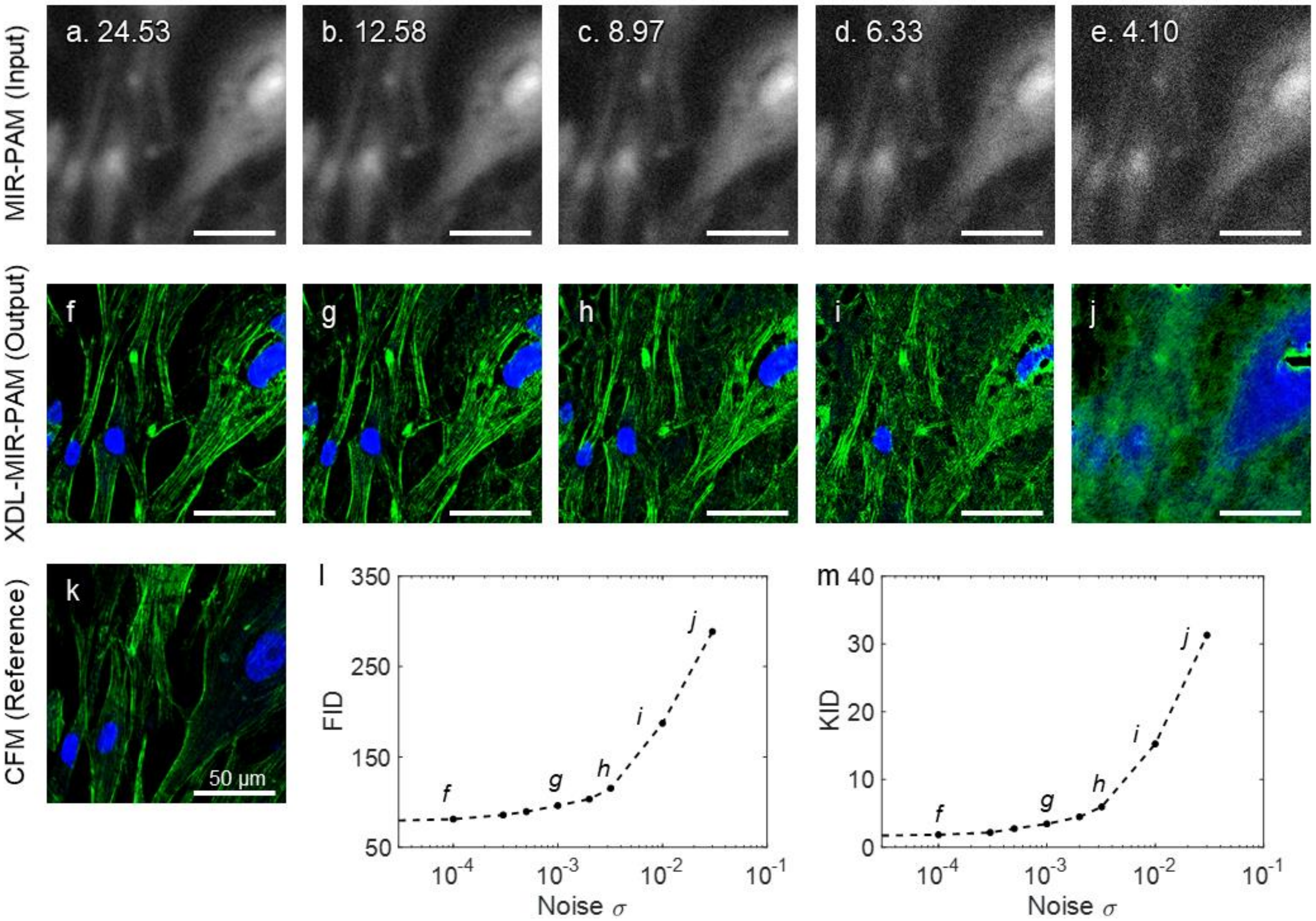

Supplementary Fig. 11 | XDL-UIDT performance according to the noise variances. a–e, Input MIR-PAM images with Gaussian noise. SNR of representative images is presented. **f–j,** Output XDL-MIR-PAM images according to the input of a–e, respectively. Results tested on the pre-built XDL framework (Net 6). **k**, The corresponding reference CFM image. Scale bar, 50 µm. **l–m**, Quantitative comparisons of FID and KID scores according to noise variances. Source data are provided as a Source Data file.

Supplementary Table. 1 | Four-fold cross-validation result

Fold	1	2	3	4	Mean
FID (↓)	100.43	106.81	100.87	105.45	103.39
KID (↓)	1.73	2.28	1.64	2.21	1.96

Supplementary References

1. Chen, C.-T. & Millero, F. J. Speed of sound in deuterium oxide relative to normal water as a function of temperature and pressure. *Journal of the Acoustic Society of America* **62**, 553–557 (1977).

2. Pleitez, M.A. et al. Label-free metabolic imaging by mid-infrared optoacoustic microscopy in living cells. *Nature biotechnology* **38**, 293-296 (2020).
