# Supplementary material for: Unsupervised inter-domain transformation for virtually stained high-resolution mid-infrared photoacoustic microscopy using explainable deep learning
Source: Nat Commun. 2024 Dec 30;15:10892. doi: 10.1038/s41467-024-55262-2 (PMC11685655; doi:10.1038/s41467-024-55262-2)
Supplement: Supplementary file 2 — Reporting Summary [file 41467_2024_55262_MOESM2_ESM.pdf]

Corresponding author(s): Chulhong Kim, Jinah Jang

Last updated by author(s): Nov 13, 2024

Reporting Summary

Nature Portfolio wishes to improve the reproducibility of the work that we publish. This form provides structure for consistency and transparency in reporting. For further information on Nature Portfolio policies, see our Editorial Policies and the Editorial Policy Checklist.

Statistics

For all statistical analyses, confirm that the following items are present in the figure legend, table legend, main text, or Methods section.

n/a	Confirmed	
☐	☒	The exact sample size (n) for each experimental group/condition, given as a discrete number and unit of measurement
☐	☒	A statement on whether measurements were taken from distinct samples or whether the same sample was measured repeatedly
☐	☒	The statistical test(s) used AND whether they are one- or two-sided<br>*Only common tests should be described solely by name; describe more complex techniques in the Methods section.*
☒	☐	A description of all covariates tested
☒	☐	A description of any assumptions or corrections, such as tests of normality and adjustment for multiple comparisons
☐	☒	A full description of the statistical parameters including central tendency (e.g. means) or other basic estimates (e.g. regression coefficient) AND variation (e.g. standard deviation) or associated estimates of uncertainty (e.g. confidence intervals)
☐	☒	For null hypothesis testing, the test statistic (e.g. F, t, r) with confidence intervals, effect sizes, degrees of freedom and P value noted<br>*Give P values as exact values whenever suitable.*
☒	☐	For Bayesian analysis, information on the choice of priors and Markov chain Monte Carlo settings
☒	☐	For hierarchical and complex designs, identification of the appropriate level for tests and full reporting of outcomes
☐	☒	Estimates of effect sizes (e.g. Cohen's d, Pearson's r), indicating how they were calculated

Our web collection on statistics for biologists contains articles on many of the points above.

Software and code

Policy information about availability of computer code

Data collection	The raw MIR-PAM data were collected using LabVIEW_2017; Image reconstruction was conducted using MATLAB_R2021b;
Data analysis	The neural networks were implemented using Python 3.10.12, CUDA 11.8.0, and PyTorch 2.1.0. TThe code is available at https://github.com/YoonChiHo/XDL_MIR_PAM_2024. Quantification for biological feature extraction was conducted using MATLAB_R2021b; Statistical analysis was conducted using GraphPad Prism 10.

For manuscripts utilizing custom algorithms or software that are central to the research but not yet described in published literature, software must be made available to editors and reviewers. We strongly encourage code deposition in a community repository (e.g. GitHub). See the Nature Portfolio guidelines for submitting code & software for further information.

Data

Policy information about availability of data

All manuscripts must include a data availability statement. This statement should provide the following information, where applicable:

- Accession codes, unique identifiers, or web links for publicly available datasets
- A description of any restrictions on data availability
- For clinical datasets or third party data, please ensure that the statement adheres to our policy

The XDL-MIR-PAM data generated in this study and test data have been deposited in the Zenodo database [https://doi.org/10.5281/zenodo.14062532]. The training datasets are available from the corresponding author upon request for research purposes.

Research involving human participants, their data, or biological material

Policy information about studies with human participants or human data. See also policy information about sex, gender (identity/presentation), and sexual orientation and race, ethnicity and racism.

Reporting on sex and gender	N/A
Reporting on race, ethnicity, or other socially relevant groupings	N/A
Population characteristics	N/A
Recruitment	N/A
Ethics oversight	N/A

Note that full information on the approval of the study protocol must also be provided in the manuscript.

Field-specific reporting

Please select the one below that is the best fit for your research. If you are not sure, read the appropriate sections before making your selection.

- [x] Life sciences
- [ ] Behavioural & social sciences
- [ ] Ecological, evolutionary & environmental sciences

For a reference copy of the document with all sections, see nature.com/documents/nr-reporting-summary-flat.pdf

Life sciences study design

All studies must disclose on these points even when the disclosure is negative.

Sample size	Cell culture was performed to provide a dataset for deep learning processing. For the training phase, about 900 sets of PAM and CFM images were used. For the test phase, about 100 image sets were used. Additional data augmentation was included in the training phase. Sample size was sufficient avoiding the overfitting and the generalization was confirmed.
Data exclusions	No data have been excluded from the experiments unless apparent failures, such as cell contamination.
Replication	Cell cultures were performed for PAM and CFM imaging to provide a dataset. Cultures for reproducibility were not performed as this was done to compare the similarity between the two imaging methods. The four-fold cross-validation was conducted in deep learning processing.
Randomization	Randomization was not a relevant feature as we were applying a uniform set. Image data were randomly divided and shuffled for unsupervised learning.
Blinding	The investigators were not blinded to allocation during experiments and outcome assessment.

Reporting for specific materials, systems and methods

We require information from authors about some types of materials, experimental systems and methods used in many studies. Here, indicate whether each material, system or method listed is relevant to your study. If you are not sure if a list item applies to your research, read the appropriate section before selecting a response.

Materials & experimental systems

n/a	Involved in the study	
[ ]	[x]	Antibodies
[ ]	[x]	Eukaryotic cell lines
[x]	[ ]	Palaeontology and archaeology
[x]	[ ]	Animals and other organisms
[x]	[ ]	Clinical data
[x]	[ ]	Dual use research of concern
[x]	[ ]	Plants

Methods

n/a	Involved in the study	
[x]	[ ]	ChIP-seq
[x]	[ ]	Flow cytometry
[x]	[ ]	MRI-based neuroimaging

Antibodies

Antibodies used	Fluorescein phalloidin (F432, Thermo Fisher Scientific, 1:40); Hoechst (H3570, Thermo Fisher Scientific, 1:1000);
Validation	Fluorescein phalloidin (F432, Thermo Fisher Scientific), https://www.thermofisher.com/order/catalog/product/kr/ko/F432;

Hoechst (H3570, Thermo Fisher Scientific), https://www.thermofisher.com/order/catalog/product/kr/en/H3570

Eukaryotic cell lines

Policy information about cell lines and Sex and Gender in Research

Cell line source(s)	HCF-c cells were purchased from PromoCell (C-12375)
Authentication	N/A
Mycoplasma contamination	The cell line was tested negative for mycoplasma contamination
Commonly misidentified lines (See ICLAC register)	N/A

Plants

Seed stocks	*Report on the source of all seed stocks or other plant material used. If applicable, state the seed stock centre and catalogue number. If plant specimens were collected from the field, describe the collection location, date and sampling procedures.*
Novel plant genotypes	*Describe the methods by which all novel plant genotypes were produced. This includes those generated by transgenic approaches, gene editing, chemical/radiation-based mutagenesis and hybridization. For transgenic lines, describe the transformation method, the number of independent lines analyzed and the generation upon which experiments were performed. For gene-edited lines, describe the editor used, the endogenous sequence targeted for editing, the targeting guide RNA sequence (if applicable) and how the editor was applied.*
Authentication	*Describe any authentication procedures for each seed stock used or novel genotype generated. Describe any experiments used to assess the effect of a mutation and, where applicable, how potential secondary effects (e.g. second site T-DNA insertions, mosiacism, off-target gene editing) were examined.*
